# Bacterial and Fungal Communities Respond Differently to Changing Soil Properties Along Afforestation Dynamic

**DOI:** 10.1007/s00248-025-02500-9

**Published:** 2025-02-06

**Authors:** Speranza Claudia Panico, Giorgio Alberti, Alessandro Foscari, Giovanni Luca Sciabbarrasi, Antonio Tomao, Guido Incerti

**Affiliations:** 1 Department of Agri-Food, Environmental and Animal Sciences, University of Udine, Via Delle Scienze 206, 33100 Udine, Italy; 2 National Biodiversity Future Center, Piazza Marina, 61, 90133 Palermo, Italy; 3 Department of Life Sciences, University of Trieste, Via Weiss 2, 34128 Trieste, Italy

**Keywords:** Soil microbiota, Space-for-time approach, Climate change, DNA metabarcoding, Soil organic carbon, α-Diversity

## Abstract

Spontaneous afforestation following land abandonment has been increasingly recognized as a nature-based solution to mitigate climate change and provide measurable benefits to biodiversity. However, afforestation effects on biodiversity, particularly on soil microbial communities, are still poorly characterized, with most previous studies focusing on artificial plantations rather than forest rewilding dynamics. Here, we assessed changes in topsoil physical–chemical properties and related dynamics of bacterial and fungal community composition and structure following spontaneous afforestation of abandoned grasslands in Northeast Italy over the last 70 years. With a space-for-time approach, we selected four chronosequences representing different successional stages: grassland, early (2000–2020), intermediate (1978–2000), and late (1954–1978). Results showed that spontaneous afforestation progressively reduced topsoil pH and total phosphorus (P), while soil organic carbon (SOC), nitrogen (N), and C:N ratio increased. Correspondingly, the overall α-diversity of the fungal community, assessed by ITS DNA metabarcoding, progressively decreased after an initial increase from grassland conditions, following substrate acidification and trophic specialization. Bacterial diversity, assessed by 16S DNA metabarcoding, was highest at the initial stages, then progressively decreased at later stages, likely limited by lower organic matter quality. Shifts of fungal community composition included an increase of ectomycorrhizal Basidiomycota linked to topsoil’s higher SOC, N, and C:N ratio. Differently, bacterial community composition responded substantially to pH, with topsoil acidity favoring Proteobacteria (Pseudomonadota) and Acidobacteria (Acidobacteriota) at the late afforestation stages. Our findings provide a first contribution to clarify how fungi and bacteria respond to spontaneous afforestation. This is particularly relevant in the context of climate change mitigation, considering the fundamental role of microorganisms in shaping soil carbon storage dynamics.

**Supplementary Information:**

The online version contains supplementary material available at 10.1007/s00248-025-02500-9.

## Introduction

Forests are of paramount importance at the global scale for climate change mitigation by sequestration and storage of atmospheric carbon (C) in above- and below-ground stocks [1, 2]. In Europe, such mitigation potential is particularly relevant due to afforestation following land abandonment, driven in the last 70 years by the progressive depopulation of mountain areas in Southern Europe, by the Common Agricultural Policy in EU Member States, and by the end of the communist regime in Central and Eastern Europe [3, 4].

The reduction of net CO_2_ emissions is the central goal of the European Green Deal, aiming for climatic neutrality by 2050 [5]. This raises the research question of whether forests can provide a cost-effective and natural toolset for enhancing C storage at the ecosystem level [6–8]. Moreover, afforestation as a nature-based solution (NbS) for climate change mitigation should also support biodiversity [9], particularly soil biodiversity, its composition, and functions. In fact, understanding microbial dynamics with spontaneous afforestation in the long term is of utmost importance to detect possible trade-offs between biodiversity conservation and climate change mitigation, under a land management perspective [10, 11].

Afforestation, as a land-use change process, progressively alters soil physicochemical properties, increasing acidity, carbon-to-nitrogen ratio (C:N), and nutrient content [12, 13]. Depending on forest type, this process could also alter litter composition and input rates [14], affecting soil organic C quality [15] and sequestration dynamics [16, 17]. These changes impact soil microbial community diversity and structure [18, 19], which play a crucial role in nutrient cycling, C sequestration, and food webs [20, 21].

Despite its significance, the effects of spontaneous afforestation on soil properties and microbial communities remain understudied, especially in the long term. Many previous studies have focused on artificial tree plantations, which often disrupt soil biota, particularly during the preparation and planting phases [22]. For instance, *Pinus tabuliformis* plantations on grasslands reduced fungal diversity but increased ectomycorrhizal (ECM) abundance [23], with nitrogen (N) availability playing a key role in driving microbial community changes. Similarly, available phosphorous (P), cation exchange capacity, and C:N ratio positively influence bacterial diversity in coniferous plantations [24]. These factors, alongside pH and other environmental variables, drive shifts in bacterial community composition. Moreover, in long-term tree plantations P and sulfur (S) availability shaped more distinctly bacterial than fungal communities [24]. Finally, in a recent study conducted in an artificial forest of *Pinus armandii*, bacterial communities were more responsive to afforestation than fungal ones [25].

Most of the previous studies dealing with microbial community shifts were limited to high-level taxonomic ranks, such as phylum level, where results often vary depending on the context. For example, in areas afforested by *Robinia pseudoacacia* and *Caragana korshinskii*, afforestation shifted the bacterial communities from Actinobacteriota- to Proteobacteria (Pseudomonadota)-dominated [26], while in afforestation by *Taxodium* in the Yangtse river basin, an increasing abundance for Nitrospirae was reported [27]. In different conditions, Acidobacteria (Acidobacteriota) can dominate soil communities, driven more by pH than by any other environmental factor [28].

The effect of single soil properties on microbial diversity is complex, especially over long-term afforestation dynamics, where stochastic processes in microbial community assembly are enhanced [29]. Conflicting findings on soil nutrient status, particularly for S and potassium (K), further complicate our understanding of afforestation impacts. Some studies suggested S increases in afforested areas due to dry deposition trapped by the forest canopy [30], whereas others indicated that S content rises due to contribution from the above-ground vegetation [31, 32]. Potassium (K) levels also vary across studies. Some research points to a decrease in soil K following afforestation, especially with *Pinus* and *Eucalyptus* plantations [12, 33], while others showed that afforestation of grasslands can enhance soil K [16, 34]. Overall, the effects of afforestation on soil nutrient content, and the resulting impact on bacterial and fungal communities, remain understudied and context-dependent and require careful consideration of local conditions, such as tree species, soil types, and geological factors, in forest management practices aimed to climate mitigation and biodiversity conservation. In this study, we selected a typical mountain area, in Northeast Italy, that underwent depopulation and land abandonment in the recent decades, leading to the spontaneous transformation of grasslands into mixed deciduous woodlands [35]. Using a space-for-time approach, we selected four afforestation stages in four replicated chronosequences spanning 70 years. In each stage, we sampled topsoil DNA for metabarcoding and analyzed associated physicochemical properties to test the following hypotheses: (i) Bacteria and fungi respond differently to changes in soil properties, depending on different trophic specialization; (ii) topsoil properties change following litter input quality and increasing canopy cover, towards high C:N ratio, more acidic pH, and low environmental variability at local scale, thus leading to less rich, but evenly distributed microbial communities.

## Materials and Methods

### Study Area

**Fig. 1:**
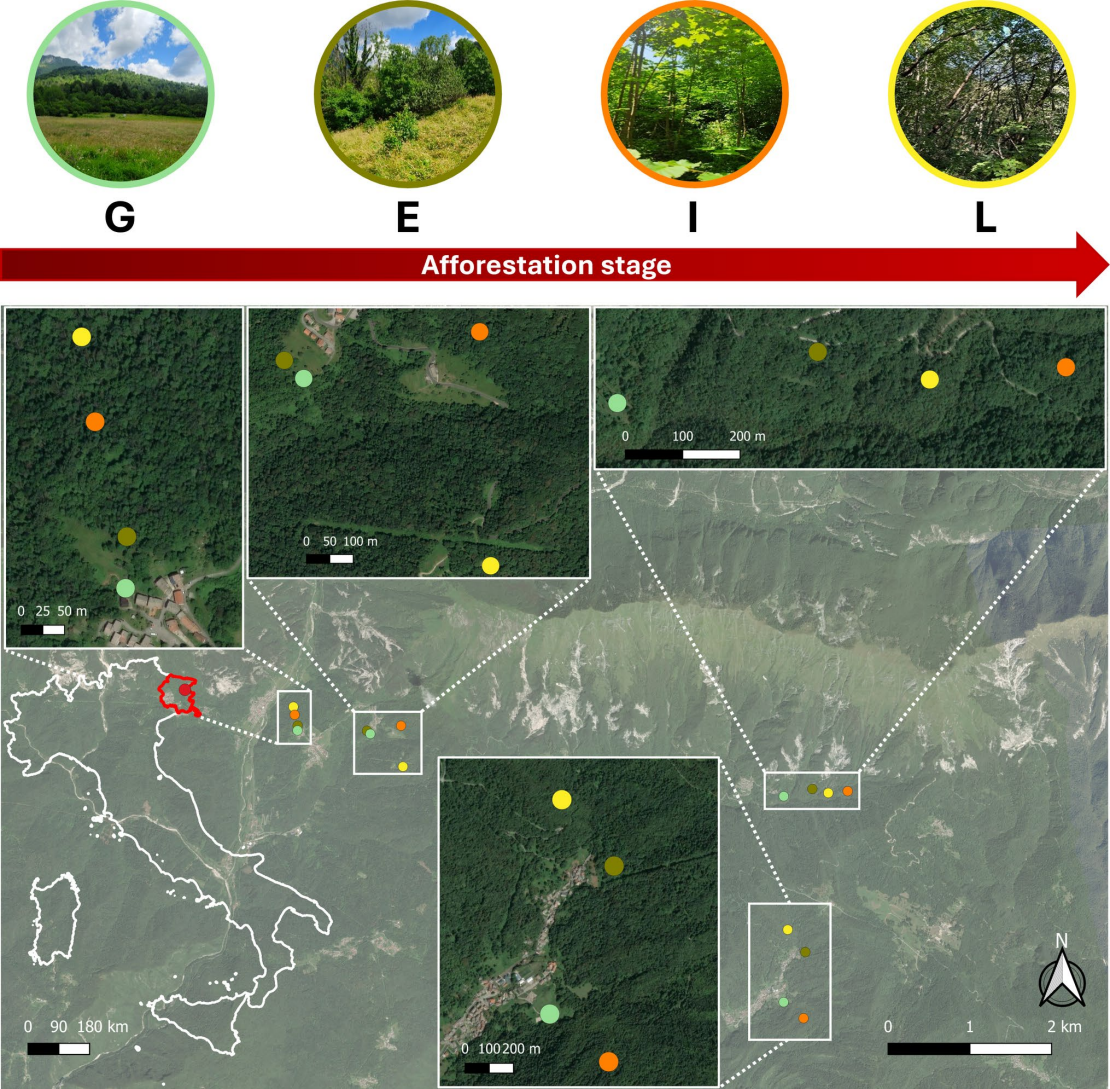
Map of the study area highlighting the location of the four chronosequences selected for topsoil sampling and, for each chronosequence, the four afforestation stages (G, grassland, green; E, early, light brown; I, intermediate, yellow; L, late, orange) with exemplificative pictures in the insets

Sampling sites are located in the municipalities of Taipana and Lusevera (Friuli Venezia Giulia Region, NE Italy, 46°31′ N, 13°30′ E) (Fig. 1), at 400–600 m a.s.l. elevation, with a mean annual temperature of 10 °C and rainfall between 2400 and 3400 mm. According to the USDA Soil Taxonomical System, local soils are classified as Cambisol, which developed from calcareous brown earths of low acidity [35]. The natural vegetation is mostly characterized by hardwood mixed stands dominated by ash (*Fraxinus excelsior* L.), sycamore (*Acer pseudoplatanus* L.), and hop-hornbeam (*Ostrya carpinifolia* Scop.), particularly abundant at late afforestation stage (Supplementary Figure S1). Small patches of *Corylus avellana* L. and *Castanea sativa* Mill. are also particularly abundant at early and intermediate stages (Supplementary Figure S1). The specific composition of the dominant vegetation cover at different afforestation stages is shown in Supplementary Figure S1. Since the early 1950s, depopulation due to socio-economic issues led to the abandonment of crops and pastures, upon which a secondary succession with natural afforestation typical of the temperate mountain forest began to establish. Thus, the survey area provides a suitable case study to investigate the effects of afforestation dynamics on soil biotic and abiotic factors.

### Sampling Design

Within the study area, four chronosequences were identified using historical georeferenced sets of digital orthophotos, showing the progressive spread of afforestation following the grassland abandonment. The sets were dated to the years 1954, 1978, 2000, and 2020. For each chronosequence, we selected four sites corresponding to the following afforestation stages: grassland (G, afforestation not yet in place in 2020), early (E, afforestation observable in 2020, but not in older orthophotos), intermediate (I, afforestation observable since 2000, but not in older orthophotos), and late (L, afforestation observable since 1978 but not in 1954 sets of orthophotos). In all sites, a randomly located, 7-m-radius circular plot was established. In each plot, in October 2023, four topsoil cores (5 cm inner diameter, 10 cm depth corresponding to the organic horizon) were collected along the perimeter of the plot at the four main cardinal directions (N, S, E, W) after litter removal and then pooled into a composite sample, resulting in 16 pooled soil samples (i.e., 4 chronosequences × 4 afforestation stages) to be used for subsequent chemical-physical analyses. In addition, three further soil cores were collected at random locations within each plot, and soil samples were kept intact to assess the average soil bulk density (BD) at each site. This sampling design explicitly takes into account the between-site and between-stages variation but not the within-plot variability of soil properties and microbial community composition, given that the latter, and its controlling factors, were widely addressed in previous literature [36].

Before pooling the samples, paying particular attention to avoid potential biological contamination, an aliquot of fine soil fraction was collected using a pre-sterilized spatula, cleaned with EtOH (37%) before and after sampling in each plot to avoid cross-contamination, and placed in 50-mL Falcon tubes, kept in an ice bag in the field, and then stored at − 20 °C in the laboratory for DNA extractions for microbial community diversity analysis. Pre-sterilized, talc-free gloves were consistently used throughout the sampling procedure to avoid possible contamination of the samples.

### Physicochemical Analyses

Composite soil samples were sieved at 2 mm to separate the fine soil fraction and afterwards submitted to the analytical procedures, from the coarse fraction, plant, and woody residues. Actual water content (*W*) was measured after drying fine fraction samples at 105 °C until reaching a constant weight. Soil pH was measured potentiometrically, using a Mettler Toledo SevenExcellence™ probe in a 1:5 fresh soil/water suspension (w/v) after 30 min shaking at 150 rpm and 6 min centrifugating at 6000 rpm. Before further analyses, soil-dried fine fractions were finely grounded (Verder Scientific S.r.l.MM500 Vario) and mixed homogeneously. SOC and total N were determined by an elemental analyzer (Elementar Italia S.r.l.; Vario Micro Tube CHNSO) after sample acidification with HCl (1:2 v/v) to remove inorganic calcium carbonates from the samples [37], and subsequently, C:N ratio was calculated. Total P, S, and K contents were analyzed by inductively coupled plasma optical emission spectrometry (ICP-OES Vista MPX, Varian Inc., Palo Alto, CA, USA), after microwave digestion with nitric (HNO_3_, 69%) and hydrofluoric (HCl, 37%) acid 1:10 v/v (USEPA 3052). All the analyses were carried out in triplicates.

### Soil DNA Metabarcoding

DNA was extracted from 200 mg aliquots of fresh, defrosted soil fine fraction for each field sample (three replicates for each plot) using the DNeasy Power Soil Kit (Qiagen) following the manufacturer’s protocol. DNA concentration was quantified, purity was checked by a spectrophotometer (Thermo Scientific PicoDrop 1000 spectrophotometer) with OD260 and OD280, and DNA quality was checked by 1% agarose gel electrophoresis. Negative controls were included in all extraction batches and were subsequently amplified to assess the presence of contaminants. For building libraries, a double-step PCR approach was used: an initial PCR amplification using locus-specific PCR primers and a subsequent amplification that integrates relevant flow-cell binding domains and unique indices (NexteraXT Index Kit, FC‐131‐1001/FC‐131‐1002). The PCRs were conducted using the following program: 3 min of denaturation at 95 °C, 25 cycles of 30 s at 95 °C, 30 s of annealing at 55 °C, 30 s of elongation at 72 °C, and a final extension at 72 °C for 5 min. PCRs were performed in triplicate using 20 µL for each sample. Libraries for bacteria were constructed using primers 341F (5′-CCTACGGGNBGCASCAG-3′) and 805R (5′-GACTACNVGGGTATCTAATCC-3′), targeting the V3–V4 bacterial 16S rRNA gene [38]. Libraries for fungi were constructed using primers ITS1 (5′-TCCGTAGGTGAACCTGCGG-3′) and ITS2 (5′-GCTGCGTTCTTCATCGATGC-3′) targeting the internal transcribed spacer ITS1 region of Eukarya [39, 40]. The amplification products were normalized to 20 ng/µL for library preparation and were sequenced on NovaSeq 6000 instrument (Illumina, San Diego, CA) using 250-bp paired-end mode based on the standard protocols by IGATech (Udine, Italy). For each library, the procedures, including sequence quality control, denoising, splicing, and chimera removal were performed after removing the sequence of unmatched primers in accordance with R dada2 package v. 1.22.0 [41]. Such procedure produced the list of amplicon sequence variants (ASVs) in each sample with great specificity, including even those sequences differing by as little as one nucleotide [42]. Then, taxonomic attribution was achieved using the SILVA (r138) and UNITE (v2019) databases for bacteria and fungi, respectively [43–45]. The taxonomic identity of each representative sequence was determined using the RDP Classifier [46]. The raw sequencing results are openly available in GenBank Archive of NCBI databases, with accession number PRJNA1159178.

Before analyzing microbial community α-diversity, the ASVs × samples datasets were standardized to equal sequencing depth among samples by rarefaction to the lowest sequencing depth (Supplementary Figure S2). Before the analysis of microbial community composition data, filtering according to relative abundances is a well-established approach for analyzing microbial community composition in eDNA datasets [47]. Several filtering options have been proposed in previous studies [48], with different arbitrary thresholds of abundance to select the features to be retained for the subsequent analysis according to the specific objectives of different studies, considering the need to remove, e.g., contamination [49], or stochasticity in β-diversity variation due to rare taxa [50]. Here, we are interested in the effects of afforestation on the community compositional transitions relevant to functions and processes at a community scale. Therefore, we focused our analysis on the most abundant fractions of bacterial and fungal assemblages, or the so-called “core-community” [48]. Therefore, both datasets for bacteria and fungi were filtered by removing all ASVs attributed to unique taxa whose total read count was lower than 0.1% of the total read count of each sample. Relative abundance was calculated by dividing the raw reads counts of each ASV in each sample by the total reads count of the sample. As such, only those ASVs with relative abundance higher than 0.1% in at least one sample were retained in bacteria and fungi datasets used for the analysis of community composition.

### Statistical Analysis

Microbial community diversity was assessed by calculating the following α-diversity metrics, based on dataset rarefied to 129,900 and 89,334 sequences per sample for bacteria and fungi, respectively (Supplementary Figure S2). Richness (i.e., total number of ASVs), Shannon’s entropy (*H*), and Pielou’s evenness (*J*) indices were analyzed using *vegan* package v. 4.4.2 [48], in RStudio version 4.2.1 [49, 50]. Although in several studies additional metrics are often considered (e.g., Chao1’s index and ACE), we excluded those relying more on specific counts of singletons or doubletons when applied to ASVs as previously suggested [51].

The effects of the afforestation stage on soil physicochemical properties and microbial community diversity metrics were tested by both parametric one-way ANOVA and non-parametric Kruskal–Wallis ANOVA, followed by pairwise comparisons using Tukey’s HSD and Mann–Whitney *U* tests for parametric and non-parametric post hoc assessments, respectively, after assessing parametric assumptions of normality and homoscedasticity by Shapiro–Wilk’s and Levene’s tests, respectively. Downstream analyses of bacterial and fungal communities were performed using the *phyloseq* package v.1.38.0 [52] and the *vegan* package v. 2.6–8 in RStudio.

Stacked bar plots were used to represent the relative abundance of the most abundant taxonomic groups. In more detail, lower ranks (i.e., family or genus) were considered for more abundant taxa, while higher ranks (i.e., phylum or class) were used for the less abundant ones.

To test the null hypothesis of no differences in fungal and bacterial community composition among different afforestation stages, we used PERMANOVA with Bray–Curtis dissimilarity index and 999 permutations to generate bootstrap *P*-values as implemented in the *RVAideMemoire* package v. 0.9–83-7 [53] using the adonis2() functions [54] in *vegan* v. 2.6–8, including in the tested model, the effect of the chronosequence, intended to express all site-specific effects (spatial variation, but also ecological variation in terms of, e.g., tree vegetation type) that of afforestation stage, and their interaction. We also performed an analysis of multivariate homogeneity (PERMDISP) after PERMANOVA using the betadisper() function to test if groups differed in dispersion [55]. Whole fungal and bacterial matrices were used as responses. A principal coordinate analysis (PCoA) was used to plot ordinations of bacterial and fungal community composition based on Bray–Curtis distance reporting all samples (*n* = 48).

In addition, principal component analysis (PCA) of the sites *x* soil properties was used to explore the multivariate relationships between soil physical–chemical features and the most abundant microbial groups (separately for bacteria and fungi), with the latter plotted as supplementary variables, directly onto the multivariate ordination biplot [56] using *ade4* version 1.7–22 and *factoextra* 1.0.7 R packages [57, 58]. Variance partitioning analysis was performed to identify the topsoil physical–chemical properties that significantly influenced bacterial and fungal community composition, using rda() function of the *vegan* package with 999 permutations sequentially adding the following predictors: soil pH, W, BD, total N, SOC, C:N, K, P, and S. Finally, Spearman correlation was used to further investigating the univariate relationships between topsoil properties and soil microbial taxa, considering |*ρ*|> 0.497 as threshold for statistically significant correlation scores, corresponding to *P* < 0.05, once corrected for multiple comparisons.

## Results

### Soil Physicochemical Properties Along the Afforestation Dynamics

Soil physicochemical properties showed significant differences among the afforestation stages (Table 1, Supplementary Table S1). In detail, water content (mean 38.6%) decreased from G to E and increased significantly at the L stage, and soil pH slightly increased from G to E and then significantly decreased from the neutral value at E down to acidic at L (Table 1). SOC and N contents showed significantly higher values at the L stage as compared to the other ones, with the rate of SOC increase with the afforestation stage, higher than that of N, resulting in a C:N ratio also significantly higher for the L (mean 10.52) compared to G and E stages (5.97 and 6.46, respectively). C:N at the I stage showed intermediate values (8.39) not significantly different from those of the other stages (Table 1). Other elemental contents showed different patterns. In the case of S, the same trend of C and N was found, with a mean of 0.90 mg kg^−1^ in L, whereas P showed the opposite trend exhibiting the highest value in G (1.64 mg kg^−1^) and significantly lower values at I stage (Table 1). The K content showed comparable values for G, E, and L stages (means of 3.44, 3.92, and 4.58 mg kg^−1^, respectively), and then it significantly increased at the I stage (means of 6.04 mg kg^−1^) (Table 1). Finally, BD of the soil fine fraction (mean 0.65 g cm^−3^) did not significantly vary among the afforestation stages.

**Table 1:** Basic soil properties at each afforestation stage (G, grassland; E, early; I, intermediate; L, late) expressed as mean ± S.E. of *W* (water content, % vol/vol), BD (bulk density, g/cm^3^), pH, N (% d.w.), SOC (% d.w.), C:N and total P, S and K contents (g/kg d.w.). Different letters indicate significant between-group differences for each soil parameter (Tukey’s post hoc test after one-way ANOVA, *P* < 0.05)

Stage		*W*	BD	pH	N	SOC	C:N	P	S	K
G	38.82 ± 7.87^ab^		0.64 ± 0.10	6.92 ± 0.37^a^	0.67 ± 0.06^b^	4.13 ± 0.81^b^	5.97 ± 0.60^b^	1.64 ± 0.61^a^	0.60 ± 0.17^b^	3.44 ± 0.8^ab^
E	31.07 ± 3.25^a^		0.69 ± 0.11	7.09 ± 0.43^a^	0.69 ± 0.07^b^	4.67 ± 1.05^b^	6.46 ± 0.68^b^	1.10 ± 0.42^ab^	0.62 ± 0.15^b^	3.92 ± 1.5^b^
I	39.17 ± 3.47^ab^		0.59 ± 0.08	6.49 ± 0.43^ab^	0.78 ± 0.07^b^	6.82 ± 1.55^b^	8.39 ± 0.99^ab^	1.02 ± 0.43^b^	0.56 ± 0.17^b^	6.04 ± 1.6^b^
L	44.02 ± 4.43^b^		0.67 ± 0.07	5.91 ± 0.25^b^	1.14 ± 0.16^a^	12.57 ± 3.95^a^	10.52 ± 2.57^a^	1.02 ± 0.42^b^	0.90 ± 0.35^a^	4.58 ± 0.3^ab^

### Taxonomic Diversity and Composition of Bacterial and Fungal Communities

The 16S rRNA and ITS primer sets were utilized to acquire a large number of high-quality sequences from the soil samples, resulting in totals of 13,001,260 16S and 10,187,610 ITS sequences, with corresponding numbers of sequences per sample ranging between 129,900 and 634,513 and between 89,334 and 356,805, respectively (Supplementary Table S3). Filtering for the rare taxa produced extremely affected ASV counts (from 148,731 to 1101 for 16S and from 103,169 to 1638 for ITS, respectively), whereas the retained ASVs corresponded to a percentage of the total number of sequences of 33.2% and 71.4% for 16S and ITS, respectively (Supplementary Table S3).

**Fig. 2:**
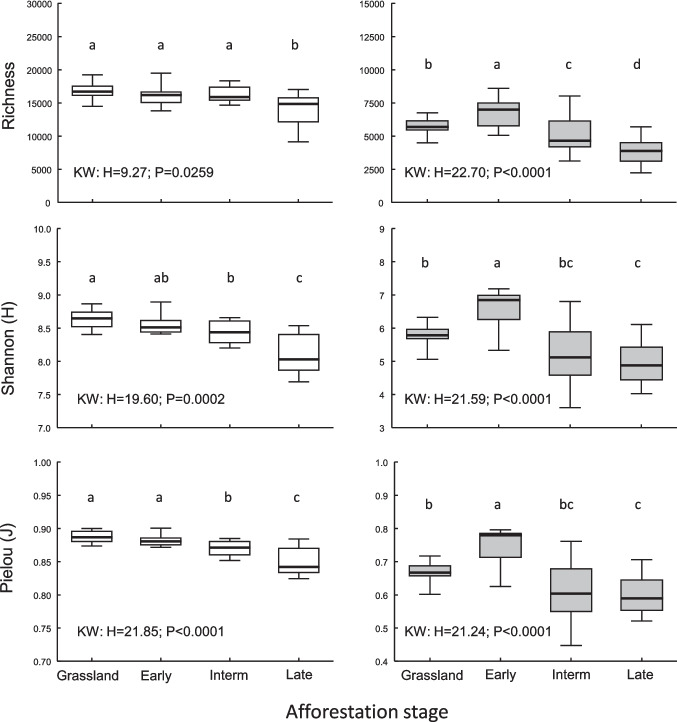
Boxplots of microbial diversity metrics (from top to bottom): richness (counts of filtered ASVs), Shannon’s (*H*) entropy, and Pielou’s evenness (*J*) for bacterial (left) and fungal (right) communities at the four different afforestation stages: grassland, early, intermediate and late. Data refer to medians, interquartile ranges (box), minimum, and maximum (whiskers) within each afforestation stage. Different letters indicate stage-dependent significant between-group differences within each plot (Mann–Whitney *U* tests at *P* < 0,05). Insets show results of Kruskal–Wallis ANOVA tests (KW statistic and associated *P*-value) for the effect of afforestation stage on each dependent variable

The afforestation stage significantly affected microbial community diversity (Fig. 2). In particular, bacterial richness was highest at the initial stages (G and E) and then progressively decreased, with a minimum at the latest stage. Equitability (Shannon’s H’) ad evenness (Pielou’s J) followed substantially the same pattern. All metrics of fungal α-diversity peaked at the early afforestation stage, following an increase from the grassland conditions, then progressively decreasing to the latest stage, where only richness was significantly lower than the preceding stage (Fig. 2). Interestingly, for fungal community α-diversity was more variable at intermediate stages, indicating higher β-diversity during the afforestation process, then converging at the latest stage. On the other hand, the variability of bacterial α-diversity was largest at the final stage, suggesting higher β-diversity in such conditions.

**Fig. 3:**
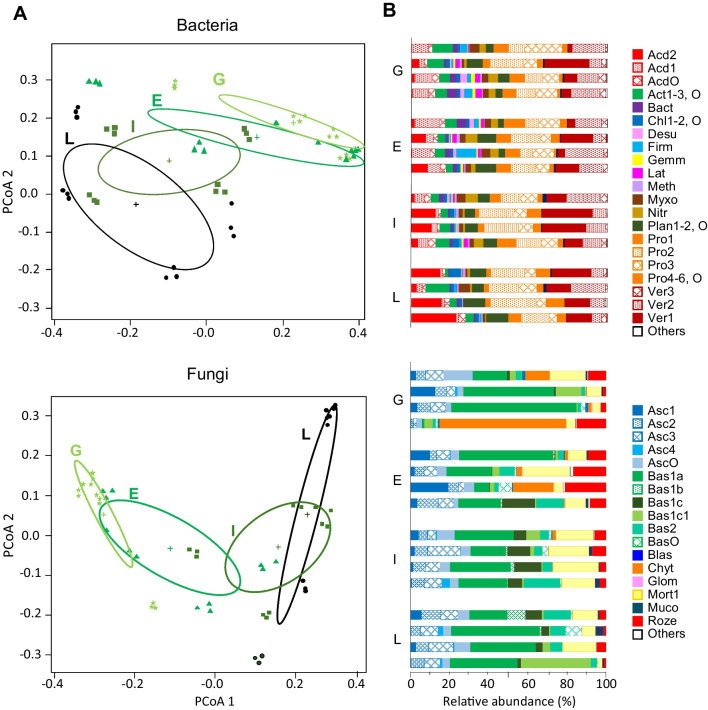
Results of principal co-ordinate analysis (**A**) showing between- and within-group compositional differences for bacteria (top) and fungi (bottom) communities. Data refer to a Bray–Curtis matrix of distances among communities sampled in four replicated chronosequences at four different afforestation stages: grassland (G, light green star), early (E, forest green square), intermediate (I, sap green triangle) and late (L, dark green circle). Community composition is also explicitly presented by barplots (**B**) showing relative abundance (%) of the most abundant taxonomic groups for bacteria (top) and fungi (bottom) at each afforestation stage and chronosequence. Taxonomic groups in the legend are labeled by acronyms to save space. Full names are in Table 4

Considering community composition, PCoA results showed that microbial communities clustered according to the afforestation stage (Fig. 3), with the spreading of within-stage groups following a trajectory corresponding to the afforestation sequence (G → E → I → L), notwithstanding a relatively high variation (i.e., symbol spreading) within the stage. As such, microbial community composition changed progressively along the afforestation dynamics consistent with what was observed for α-diversity indices. Stage-dependent differences were confirmed by the PERMANOVA results (Table 2), showing that the fixed effect of the afforestation stage in community composition was highly significant for both bacteria (*P* = 0.001) and fungi (*P* = 0.001). Correspondingly, pairwise compositional differences among afforestation stages were significantly different only when comparing the initial (G) vs. the final (L) stages (Supplementary Table S2, *P* = 0.049 and *P* = 0.015 for bacteria and fungi, respectively). The remaining pairwise comparisons, involving early and intermediate afforestation stages, did not show significant differences (Supplementary Table S2), likely due to the relatively large within-stage variability, corresponding to the within-group dispersion around the respective centroids in the PCoA (Fig. 3). Such variability at the within-stage scale possibly indicates significant variation of β-diversity along the afforestation process. Indeed, site-dependent effects were in turn statistically significant (*P* = 0.030 and *P* = 0.030 for bacteria and fungi, respectively, Table 2). Finally, a significant interaction term in both PERMANOVA models for bacteria and fungi (Table 2) clearly indicated that compositional shifts along the afforestation dynamics proceeded differently at different sites. When compositional variation was tested with topsoil properties as predictors, interesting differences between bacterial and fungal communities emerged. In particular, the results of variance partitioning analysis, in addition to a predominant and generalized effect of pH, showed a significant contribution of SOC and total N to bacterial community composition, whereas the composition of the fungal community was only affected by the ratio of C and N contents, but not by their individual contributions (Table 3). Interestingly, residual variation, not explained by the tested predictors, was relatively low, likely attributable to the restrictive filtering procedures that excluded rare taxa from the analysis.

**Table 2:** Results of PERMANOVA testing for the effects of site/chronosequence (CHR, random effect) and afforestation stage (ST, fixed effect with 4 levels: G, E, I, L, see main text) and their interaction on bacteria and fungi community taxonomic composition. Data refer to effect type, degrees of freedom (Df), sum of squares (SS), *F* and associated *P*-value (bootstrap with 999 permutations), and model *R*^2^

Effect	Df	SS	*F*	*P*	*R*^2^
Bacteria					
Chronosequence (CHR)	1	0.267	2.703	0.030	0.040
Afforestation stage (ST)	3	1.746	5.877	0.001	0.262
CHR × ST	3	0.674	2.267	0.016	0.101
Residual	40	3.962			0.595
Fungi					
Chronosequence (CHR)	1	0.595	5.132	0.030	0.060
Afforestation stage (ST)	3	3.216	6.918	0.001	0.268
CHR × ST	3	1.761	3.789	0.013	0.147
Residual	40	6.199			0.517

**Table 3:** Variance partitioning of community composition as resulting from redundancy analysis of bacteria and fungi (see the “Materials and Methods” section). Total variance explained by the tested predictors and the unexplained residuals is shown as long as the list of predictors with their individual contributions to explained variance (raw and percentage values). For each predictor, *P* indicates the statistical significance of *F* test with 999 permutations. Significant *P*-values (*P* < 0.05) are marked with italic font

	Bacteria			Fungi		
Predictor	Exp. variance	%	*P*	Exp. variance	%	*P*
pH	9.6078	36.7	*0.0010*	2.6019	9.9	*0.010*
*W*	0.9363	3.6	0.6700	0.8061	3.1	0.693
BD	2.4138	9.2	0.0620	1.6574	6.3	0.110
SOC	3.6893	14.1	*0.0499*	1.8045	6.9	0.114
N	2.7282	10.4	*0.0100*	1.6406	6.3	0.132
C:N	2.2242	8.5	0.1080	1.7969	6.9	*0.010*
K	1.298	5.0	0.4250	1.2304	4.7	0.307
P	2.0069	7.7	0.1480	1.1318	4.3	0.384
S	1.3037	5.0	0.4350	0.8844	3.4	0.629
Total	26.2082	77.1		13.554	67.8	
Error	7.7918	22.9		6.446	32.2	

Considering single taxonomic groups, the dominant bacterial phyla were Acidobacteria, Actinobacteriota, Proteobacteria, and Verrucomicrobiota with an overall increasing abundance along the afforestation process: 73% at G and E stages, 79% at I stage, and 81% at L stage (Fig. 3). In more detail, all these phyla increased from G to L stage, while Actinobacteriota decreased from 7.5 to 5.3%. (Fig. 3). Rarer phyla such as Chloroflexi and Planctomycetota increased by 0.8% and 3.5% from the G to the L stage, respectively (Fig. 3). Differently, Nitrospirota, Firmicutes, and Latescibacteriota decreased from G to L stage with a final relative abundance of 1.7%, 1.9%, and 2%, respectively (Fig. 3). At lower taxonomic rank, more interesting trends emerged. For example, within Acidobacteria, the class Acidobacteriae was dominant at the G stage but progressively decreased at later stages, whereas Vicinamibacteria showed the opposite trend (Fig. 3). Within Proteobacteria, the class Gammaproteobacteriae decreased by afforestation and Alphaproteobacteria, particularly the Xanthobacteraceae family, increased (Fig. 3). Within Verrumicrobiota, the Chthoniobacteraceae and Xiphinematobacteraceae families showed opposite patterns, with the former increasing and the latter decreasing along the afforestation dynamics (Fig. 3).

The dominant fungal phyla were Ascomycota and Basidiomycota (Fig. 3). Their cumulative relative abundance progressively increased with afforestation age, with the following sequence of values: 63% (at G stage), 67% (E), 80% (I), and 89% (L). However, these two phyla showed a different response to afforestation dynamics. Ascomycota did not vary consistently, showing mean relative abundance between 20 and 30% from G to L stages at all sites, with the lowest and highest exceptions of stage G at site 4 (7%) and stage E, at site 3 (40%). On the other hand, Basidiomycota progressively increased from 44.1 at G to 69.4% at L stages. Within Basidiomycota, the order Agaricales decreased along the afforestation dynamics, whereas Boletales, Cantharellales, and Russulales increased (Fig. 3). Considering the remaining phyla (Fig. 3), Chytridiomycota showed a mostly site-dependent pattern; Rozellomycota showed highest abundance at the E stage then decreasing at later stages; Mortierellomycota showed highest abundance at either E or I stage; Mucoromycota showed a mean abundance of 2% at L stage (with a peak of 4.8% at site 3) and < 1% at earlier stages. Finally, Blastocladionmycota showed an opposite pattern, with a mean abundance of 1.4% at the G stage (with a peak of 2.9% at site 1), less than 1% at later stages, and not being found at L one.

### Bacteria and Fungi Response to Soil Properties Along the Afforestation Dynamics

**Fig. 4:**
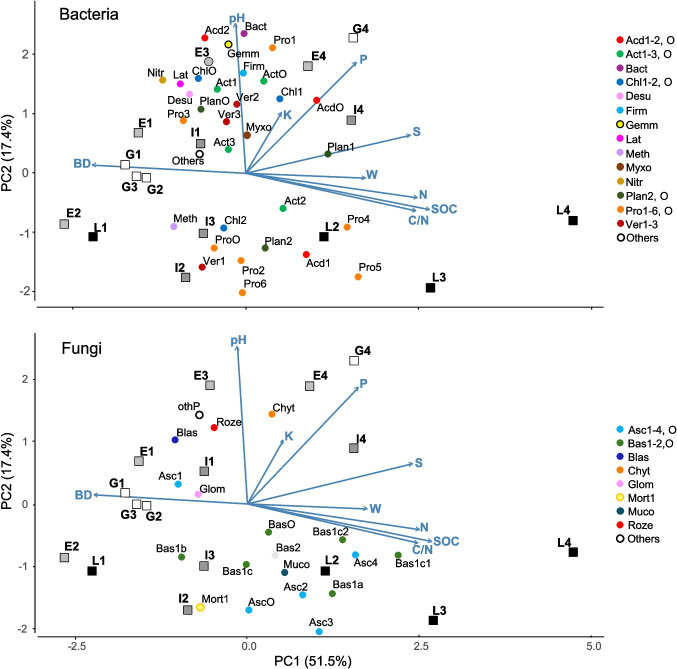
Principal component analysis (PCA) biplot showing the spatial distribution of soil samples (*n* = 16) in the ecological space defined by soil properties (blue vectors, BD, pH, *W*, SOC, C:N, N, K, P, and S used as supplementary variables). Samples differ by afforestation stage (white, grassland, G; light grey, early, E; dark grey, intermediate, I; black, late, L). Most abundant taxonomic groups of bacteria (top) and fungi (bottom) are also plotted as supplementary variables (see “Materials and Methods” section) and indicated with colors corresponding to phyla and labeled with acronyms indicating lower rank groups within the respective phylum. Full names are in Table 4

The results of the PCA showed two main components (PC1 and PC2) accounting for 68.9% of the total data variance (Fig. 4). PC1 was positively associated with SOC, N, C:N, W, and nutrient content (S, P, and, though with lower vector loading, K) and negatively to BD. Topsoil pH was the variable most associated with PC2, with further contributions by P, K, and, to a lesser extent, S, indicating a gradient of increasing organic matter content from left to right and increasing soil acidity and lower nutrient content from top to bottom (Fig. 4). The distribution of soil samples confirmed in some cases a within-stage similarity of soil properties. In particular, most of the G samples clustered at the leftmost of PC1, hence characterized by high bulk density and low organic matter and nutrient content. Differently, L samples were sparsely placed along the PC1, as related to their variable bulk density and organic matter and nutrient contents, but consistently at the bottom of PC2, therefore sharing acid pH conditions. E and I samples were variably distributed in the bi-dimensional space, therefore showing variable soil properties, suggesting the age-dependent trajectory that, starting from a common pattern at the G stage, led to different conditions in different sites at the L stage (Fig. 4).

By plotting the most abundant taxonomic groups of bacteria and fungi onto the physical–chemical biplot space as supplementary variables (Fig. 4), the multivariate relationships between topsoil features and microbiota emerged. Indeed, the bacterial communities were placed into two main assemblages along the PC2, affected by soil pH and nutrient content.

**Fig. 5:**
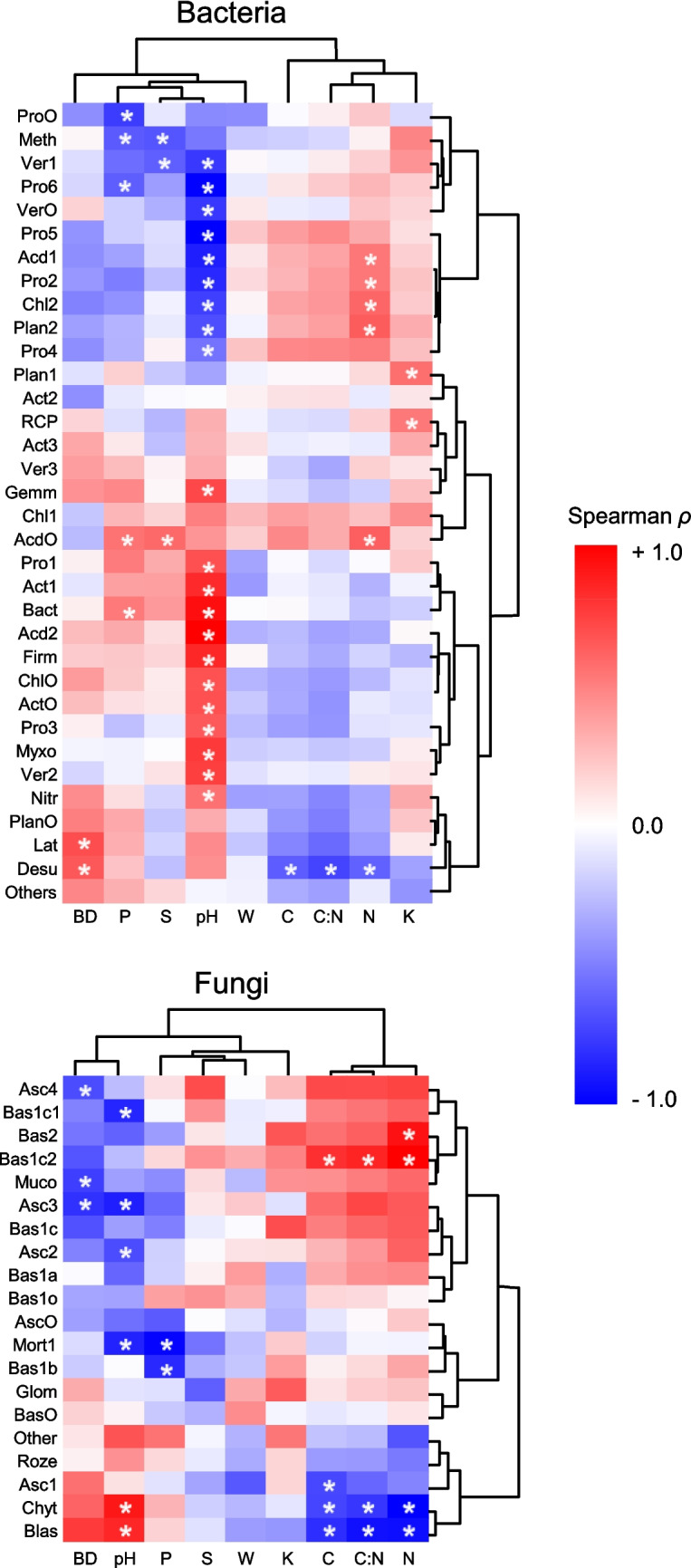
Heatmap based on Spearman’s *ρ* correlation scores calculated between the relative abundance of main microbial groups of bacteria (top) and fungi (bottom) in topsoil samples and the abiotic properties (BD, pH, *W*, SOC, C:N, N, K, P, S) of the same samples. Dendrograms show groups of taxa and chemical-physical parameters showing similar distribution in the samples. Asterisks (*) indicate statistically significant correlation scores (*ρ* >|0.497|, *P* < 0.05)

As shown by the results of univariate Spearman correlation analysis (Fig. 5), the first assemblage, positively associated to soil pH, included Acidobacteria, Actinobacteriota other than Actinobacteria and Thermoleophilia, Bacteroidota, Chloroflexi other than Chloroflexia and Ktedonobacteria, Firmicutes, Myxococcota, Verrucomicrobiota such as Xiphinematobacteraceae, Gammaprotobacteria, and Rhizobiales other than Xanthobacteraceae (Fig. 4, Supplementary Table 4). The second assemblage included acidophilic taxonomic groups, with abundance negatively correlated to soil pH (Fig. 5), and variable preferences for different conditions of soil bulk density and organic matter content, such as Actinobacteria; Ktedonobacteria; Planctomycetes; Alphaprotobacteria such as Xanthobacteriaceae, Micropepsaceae and Acetobacteriaceae, and Chthoniobacteraceae; and Verrucomibrobiota other than Verrucomicrobiae (Fig. 4, Supplementary Table 2). Further significant correlations among soil properties and bacterial groups were found (Fig. 5), as in the cases of soil K content, positively influencing the abundance of the phylum RCP2-54 and the class Phycisphaerae of Planctomuycetota; total P content enhanced Bacteroidota, Xanthobacteraceae, Ktedonobacteria, Planctomycetes, and Acidobacteria, while negatively affecting some groups of Proteobacteria and Methylomirabilota. The latter, as well as Chthoniobacteraceae, were also negatively affected by S content. Finally, SOC, N content, and C:N inhibited Desulfobacterota (Fig. 5, Table 4).

**Table 4:** Label code for bacteria and fungi taxa with phylum, class, order, and family reported in Figs. 3, 4, and 5

Label	Phylum	Class	Order	Family
Bacteria				
Acd1	Acidobacteria	Acidobacteriae	-	-
Acd2	Acidobacteria	Vicinamibacteria	-	-
AcdO	Acidobacteria	Other_classes	-	-
Act1	Actinobacteriota	Acidimicrobia	-	-
Act2	Actinobacteriota	Actinobacteria	-	-
Act3	Actinobacteriota	Thermoleofilia	-	-
ActO	Actinobacteriota	Other_classes	-	-
Bact	Bacteroidota	-	-	-
Chl1	Chloroflexi	Chloroflexia	-	-
Chl2	Chloroflexi	Ktedonobacteria	-	-
ChlO	Chloroflexi	Other_classes	-	-
Desu	Desulfobacterota	-	-	-
Firm	Firmicutes	Bacilli	-	-
Gemm	Gemmatimonadota	-	-	-
Lat	Latescibacterota	-	-	-
Meth	Methylomirabilota	-	-	-
Myxo	Myxococcota	-	-	-
Nitr	Nitrospirota	Nitrospiria	-	-
Plan1	Planctomycetota	Phycisphaerae	-	-
Plan2	Planctomycetota	Planctomycetes	-	-
PlanO	Planctomycetota	Other_classes	-	-
Pro1	Protobacteria	Gammaproteobacteria	-	-
Pro2	Protobacteria	Alphaprotobacteria	Rhizobiales	Xanthobacteraceae
Pro3	Protobacteria	Alphaprotobacteria	Rhizobiales	Other_families
Pro4	Protobacteria	Alphaprotobacteria	Reyranellaceae	-
Pro5	Protobacteria	Alphaprotobacteria	Micropepsaceae	-
Pro6	Protobacteria	Alphaprotobacteria	Acetobacteraceae	-
ProO	Protobacteria	Alphaprotobacteria	Other_classes	-
RCP	RCP2-54	-	-	-
Ver1	Verrucomicrobiota	Verrucomicrobiae	Chthoniobacterales	Chthoniobacteraceae
Ver2	Verrucomicrobiota	Verrucomicrobiae	Chthoniobacterales	Xiphinematobacteraceae
Ver3	Verrucomicrobiota	Verrucomicrobiae	Pedosphaerales	-
VerO	Verrucomicrobiota	Other_classes	-	-
Others	Other phyla	-	-	-
Fungi				
Asc1	Ascomycota	Archaeorhizomycetes	-	-
Asc2	Ascomycota	Eurotiomycetes	-	-
Asc3	Ascomycota	Leotiomycetes	-	-
Asc4	Ascomycota	Saccharomycetes	-	-
AscO	Ascomycota	Other_classes	-	-
Bas1a	Basidiomycota	Agaricomycetes	Agaricales	-
Bas1b	Basidiomycota	Agaricomycetes	Boletales	-
Bas1c	Basidiomycota	Agaricomycetes	Cantharellales	-
Bas1c1	Basidiomycota	Agaricomycetes	Russulales	-
Bas1c2	Basidiomycota	Agaricomycetes	Sebacinales, Thelophorales	-
Bas1o	Basidiomycota	Agaricomycetes	Others	-
Bas2	Basidiomycota	Tremellomycetes	-	-
BasO	Basidiomycota	Other_classes	-	-
Blas	Blastocladiomycota	-	-	-
Chyt	Chytridiomycota	-	-	-
Glom	Glomeromycota	-	-	-
Mort1	Mortierellomycota	Mortierellomycetes	-	-
Muco	Mucoromycota	-	-	-
Roze	Rozellomycota	-	-	-
Others	Other_phyla	-	-	-

In the case of fungal communities, the PCA results (Fig. 4) highlighted three clusters showing different patterns of association with soil properties. The first cluster included fungi placed in the right-bottom quadrant in the biplot, showing positive and negative factorial scores along the PC1 and PC2, respectively. As such, these fungi were mostly associated with acidic soil conditions and high organic matter content (Fig. 4), as confirmed by the univariate correlation patterns (Fig. 5) showing the positive effect of SOC, C:N, and N content on specific groups of Basidiomycota (Table 4), such as the Agaricomycetes belonging to orders Sebacinales, Thelephorales, and Russulales, with the latter also being negatively associated to pH, hence showing acidophilic response, along with the Ascomycota classes Eurotiomycetes and Leotiomycetes (Fig. 4). Other fungi belonging to the first cluster included Mucoromycota and Saccharomycetes, which were negatively associated to soil bulk density (Fig. 5, Table 4). A second cluster of acidophilic fungi, unrelated to organic matter variables (Fig. 5), and then placed at the bottom-center of the PCA biplot (Fig. 4), included, among others, Boletales and Mortierellomycetes (Table 4), which, interestingly, were also negatively associated to P (Fig. 5). Finally, the third cluster of fungi, showing positive scores along the PC2 and then placed at the top of the PCA biplot (Fig. 4), included, among others, Archaeorhizomycetes, Chytridiomycota, and Blastocladiomycota (Table 4), all negatively associated to SOC, with the two latter groups also negatively associated to N and C:N, and positively to pH.

## Discussion

### Effect of Afforestation on Soil Properties

Our findings clearly showed that spontaneous grassland afforestation affects soil properties such as pH, SOC, and nutrient content. Specifically, soils in the later stages of afforestation, compared to those in grasslands, were more acidic, with increased SOC, N, C:N, total S, and decreased P.

A decline in soil pH is a well-documented outcome of afforestation, primarily due to the changes in litter fall occurring along the successional dynamics [59]. In addition to a progressive increase in the amount of litter yearly deposited to the forest floor, at least during the first decades of afforestation [60], the chemical quality of litter materials becomes progressively more acidic, as compared to that of grasses that persist the initial successional stages. Moreover, it was highlighted that afforestation globally leads to soil acidification and can significantly enhance soil nutrient availability [59], although the dynamics of N and P can vary depending on factors such as ecozone, forest type, tree species, and climate [61]. In this study, the significant reduction in soil P following afforestation is a known feature of the temperate forest zone [61]. Indeed, in temperate regions, and particularly at low temperatures, plants may preferentially use existing labile P forms over newly mineralized ones, ultimately consuming the available P pool [62]. Additionally, trees tend to have a higher demand for P than grasses, often resulting in a decrease in soil P content following grassland afforestation [63].

### Resource Availability and Trophic Specialization Limit Fungal Community Diversity

Substantial shifts of topsoil chemical properties influenced the composition and diversity of soil microbial communities, but with different responses for bacteria and fungi along the afforestation process. Fungal community diversity progressively decreased during plant successional dynamics, following grassland afforestation. These findings suggest that afforestation can lead to progressively more homogeneous edaphic conditions at the late stage, hence to a narrower range of niches, which in turn could drive to a reduction of the total number of taxa, increasing local extinction by competitive exclusion, and hence to a more even resource distribution by niche partitioning among the survivors. In other words, the increasing acidity and recalcitrance of litter inputs [59] could reduce the total pool of fungal species to those capable to either feed upon intrinsically limiting substrate or to undertake trophic interactions with tree roots, as in the case of mycorrhizal fungi, which we found thriving at the late afforestation stage. Accordingly, the progressive increase in SOC and nutrient content could result in increased overall resource availability, reducing interspecific competition at the late stages and enhancing the relative abundance of all the (trophically competent) fungi. Previous observations are only partially consistent with our explanation, as papers on soil microbiome changes along afforestation dynamics refer to remarkably different conditions with respect to our study in terms of ecoregion, substrate type, tree species, and time span (e.g., [23–25, 29, 64, 65]). In fact, contrary to our results, some previous studies [23, 64, 65] found bacterial and fungal richness increases following artificial reforestation in different areas of China. In particular, Wang et al. [23] related their findings to large tree root growth enhancing soil porosity, which fosters the accumulation of root exudates, and soil aeration ultimately boosts microbial metabolism. Differently, consistent with our findings, Panico et al. [66] reported that the increase of SOC, N, and C:N ratio creates favorable conditions for the growth of fungal communities capable of decomposing recalcitrant organic matter.

### Resource Quality, More than Quantity, Controls Soil Bacterial Community Diversity

Bacterial communities underwent remarkable changes especially at the late afforestation stage, with significant compositional differences between different stages and sites. Such a pattern indicates that bacterial community diversity is not directly enhanced by overall resource availability, in terms of amounts of soil organic matter and nutrients (as we observed at the late afforestation stage). Rather, it is reasonable to infer that the highest bacterial diversity at early-intermediate afforestation stages is sustained by the wider range of quality of available resources, in turn derived from the molecular diversity of the litter input [67]. Indeed, at early and intermediate successional stages, the highest plant diversity can be maintained by the persistence of some of the open-habitat species occurring at the pre-afforestation stage, as well as the establishment of the first individuals of the late-successional forest species, in addition to generalist species [68]. A high plant diversity, not (only) taxonomically but in terms of leaf traits, should also correspond to the large molecular diversity of the leaf litter input [69] and hence of the dissolved organic matter (DOM) that leaches into the soil from the litter layer. In this respect, it is well known that microbial life in the soil is fuelled by dissolved organic matter that leaches from the litter layer [70] and that the soil bacterial community specializes towards the molecular type of litter source and its state of decomposition [71, 72]. However, the causal role of soil pH cannot be excluded, at least because it directly affects the molecular diversity of SOC, as differentially influencing the decomposition rates of different C pools. At this stage, we are aware that our explanatory hypothesis is somehow speculative, as we did not characterize soil organic matter in our soil samples in terms of molecular diversity. However, this specific issue will be addressed in a further study, with the molecular quality of soil organic matter assessed by 13C-CPMAS-NMR [73, 74].

### Compositional Shifts of Microbial Communities Following Afforestation

Core community composition distinctly differed between grassland and late-stage sites, while the early and intermediate afforestation stages exhibited more compositionally similar assemblages. This directly reflects the progressivity in the changes in topsoil properties. In our research, Proteobacteria, Acidobacteria, and Verrucomicrobiota were more abundant in afforested soils compared to grassland, and their prevalence increased with the afforestation stage, which is consistent with some previous findings from the forest soil environment [75, 76]. Among the topsoil properties more associated with bacteria, pH, SOC, and N were the most predictive factors influencing the overall community composition and structure, as well as controlling the abundance of single taxonomic groups, as in the cases of Verrucomicrobia, Bacteroidetes, and Acidobacteria, indicating that lower-rank taxa within these groups likely share similar ecological traits [77]. Similarly, oligotrophic (low nutrient, *r*-strategy) or copiotrophic (high nutrient, K-strategy) traits may be deeply conserved among soil bacteria, as emerging at high taxonomic rank [78].

Overall, the observed changes in composition likely reflect the response of the core community to broader ecosystem succession and vegetation shifts, as plant inputs (leaf litter, root biomass, and exudates) vary by vegetation type and have a strong influence on the microbial response [71]. For example, we observed an increase in Alphaproteobacteria and a decrease in Actinobacteria, which aligns with a transition from grassland to forest soils [79].

Moreover, during the afforestation, low soil pH and C:N ratio explained significant fractions of the compositional variance of the fungal communities, also showing significant associations with single taxonomic groups. This finding highlights the dependence of soil fungal development on specific substrate types in terms of molecular quality, as C:N is a well-known indicator of soil organic matter quality and decomposability [80] and therefore a critical factor for niche-driven fungal community assembly. In more detail, the predominant fungal phyla in our dataset were Ascomycota and Basidiomycota, which are the largest groups in both grassland and forest ecosystems [81, 82]. Ascomycota were consistently abundant before and after the grassland-to-forest transition. Their stability irrespective of the above-ground condition could be due to the stress-resistant and highly competitive nature of most Ascomycota [83]. On the other hand, Basidiomycota have been frequently reported as the dominant fungal group in forest soils [84, 85]. In our study, their relative abundance was highest at the late stages. Basidiomycetes fungi are effective decomposers, particularly thriving on dead wood or litter. In fact, the lignin-rich environment of forest soils likely explains the high abundance of Basidiomycota in these sites [86]. These fungi are capable of degrading plant and animal residues, and some species even cause wood decay [23]. Additionally, certain Basidiomycota are known to withstand extreme environmental conditions like high temperatures, cold, drought, and UV radiation [81]. Given that early-stage afforested sites lack mature tree cover, which normally buffers against fluctuating conditions and reduces direct UV exposure, these fungi may have a competitive edge in such challenging environments. Among more specific taxa, in addition to saprotrophs, we observed increases in tree-associated ectomycorrhizae (e.g., Boletales, Cantharalleles, and Russulales [87]). These are well-known ECM responsive to P and N [88]. As such, in temperate deciduous forest soils, they can play important roles in nutrient cycling, often making up a similar or even greater fraction of the fungal community compared to saprotrophs [89].

We acknowledge that our results may possibly be regarded as preliminary, as the intrinsic limited sample size prevented a proper assessment of microbiome compositional variation at all the explored spatial levels. Particularly, while afforestation stage-dependency and between-site variability were disentangled by our PERMANOVA model, further within-site heterogeneity at a small spatial scale was not clarified. However, we considered it of major interest to address larger-scale effects, also considering that these broader studies are ongoing, aiming to clarify the benefits of natural afforestation and associated biodiversity patterns at an international scale.

## Conclusion

This study highlighted that natural afforestation following grassland abandonment significantly alters topsoil physicochemical properties and soil microbes, at the core community scale. The process led to progressive enrichment of SOC and nutrients and soil acidification driven by increased plant litter input and changes in organic matter quality. Soil content of K and P varied based on environmental factors such as forest type and afforestation age. Bacterial communities were decisively influenced by pH, with acidic conditions favoring Proteobacteria (Pseudomonadota) and Acidobacteriota while limiting other bacteria, hence resulting in a progressive decline of α diversity at a late afforestation stage. Fungal communities, after an initial enhancement compared to grassland conditions, showed declining α diversity, with ectomycorrhizal Basidiomycota prevailing at later stages. These findings provide a first contribution to the understanding of microbial responses to spontaneous afforestation, a topic interrelated with biogeochemical cycles, and then of utmost relevance in the frame of nature-based solutions for climate change mitigation.

## Supplementary Information

Below is the link to the electronic supplementary material.

Supplementary file1 (PDF 441 KB)

## Data Availability

No datasets were generated or analyzed during the current study.
