# Supplementary material for: Bacterial and Fungal Communities Respond Differently to Changing Soil Properties Along Afforestation Dynamic
Source: Microb Ecol. 2025 Feb 6;88(1):2. doi: 10.1007/s00248-025-02500-9 (PMC11799125; doi:10.1007/s00248-025-02500-9)
Supplement: Supplementary file 1 — Supplementary file1 (PDF 441 KB) [file 248_2025_2500_MOESM1_ESM.pdf]

Bacterial and fungal communities respond differently to changing soil properties along afforestation dynamics

Speranza Claudia Panico[1,2,*], Giorgio Alberti[1], Alessandro Foscari[1], Giovanni Luca Sciabbarrasi [1,3], Antonio Tomao[1], Guido Incerti[1]

[1]Department of Agri-food, Environmental and Animal Sciences, University of Udine, via delle Scienze 206, 33100 Udine, Italy.

[2]National Biodiversity Future Center, Piazza Marina, 61 90133 Palermo – Italy.

[3]Department of Life Sciences, University of Trieste, via Weiss 2, 34128 Trieste, Italy.

*Corresponding author:
Speranza Claudia Panico
speranza.panico@uniud.it
Department of Agri-food, Environment and Animal Sciences
University of Udine
via delle Scienze 206
33100 Udine
Italy

SUPPLEMENTARY INFORMATION

Supplementary Figure S1. Basal area of forest stands along the afforestation dynamics.

Supplementary Figure S2. Rarefaction curves from bacteria and fungi datasets.

Supplementary Table S1. Results of one-way ANOVA and Tukey's HSD post hoc tests on soil physico-chemical properties.

Supplementary Table S2. Pairwise comparisons among microbial community compositions from different afforestation stages.

Supplementary Table S3. Counts of ASVs and sequences in bacteria and fungi datasets.

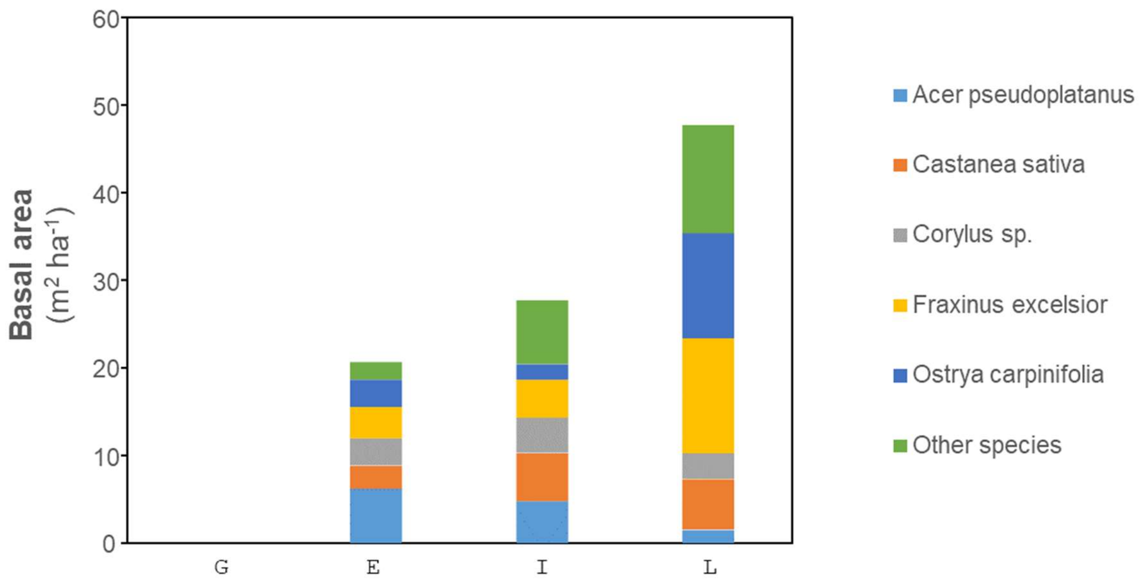

Supplementary Figure S1. Basal area of forest stands along the afforestation dynamics. Stacked barplot of basal area of main tree species across the four afforestation stages: Early (E), Intermediate (I), and Late (L). For each species, data refer to mean basal area of four replicated sampling plots.

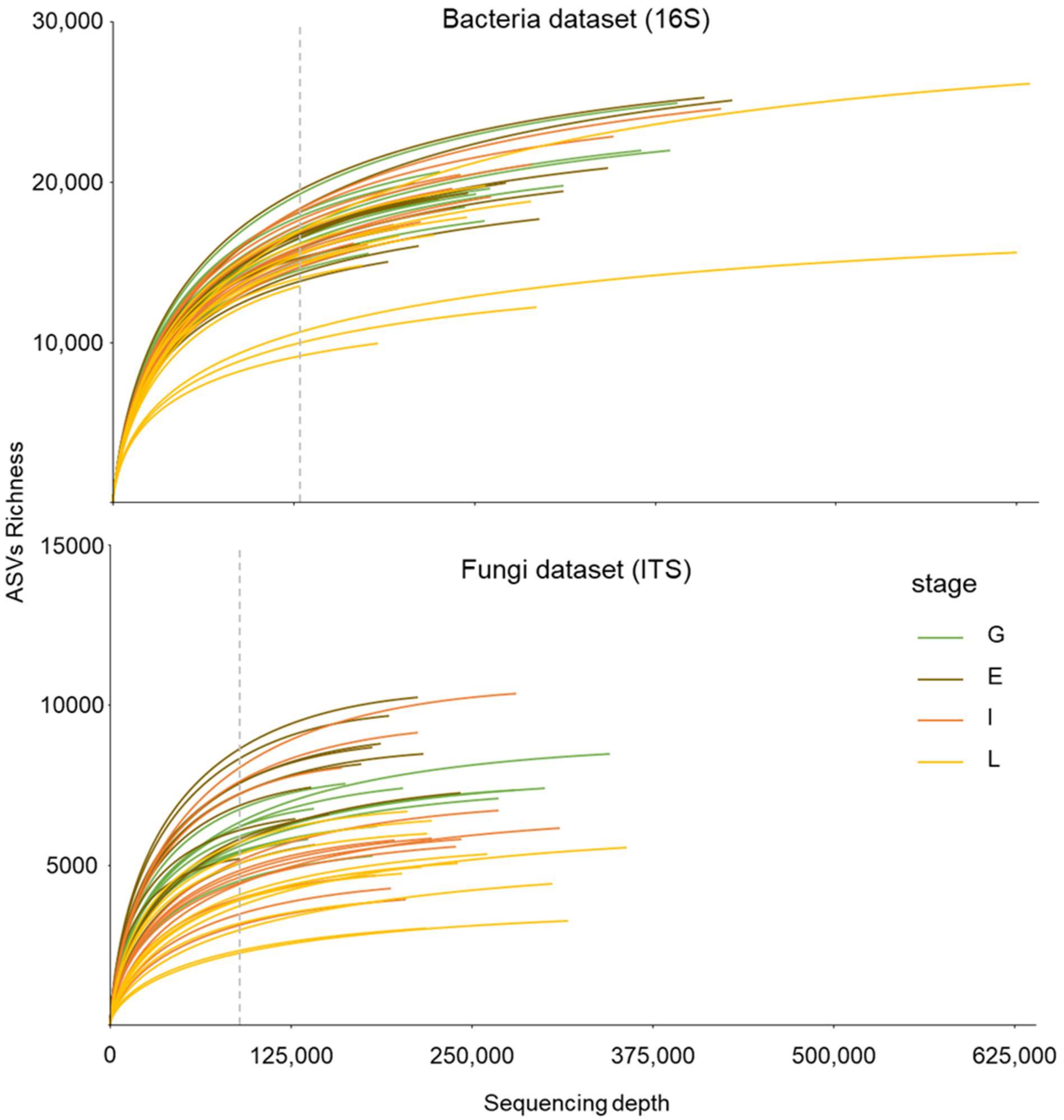

Supplementary Figure S2. Rarefaction curves from bacteria and fungi datasets. Data refer to counts of ASVs for each sample (N=48, symbolized according to afforestation stage) as a function of sequencing depth, up to realized depth. At lower depth values (every 1000 sequences), Y-values for each sample were calculated as means of bootstrapping subsamples with 100 iterations for each sequencing depth value. Vertical dashed lines indicate the levels of rarefaction selected for the alpha-diversity analysis (see main text), corresponding to the minimum observed values of 129900 and 89334 for bacteria and fungi, respectively.

Supplementary Table S1. Results of one-way ANOVA (DF, degrees of freedom, SS: Sum of squares, F statistics and associated P-values) and Tukey's HSD post hoc tests for the effect of afforestation stage (G-grassland, E- early, I-intermediate, L-late) on soil physico-chemical properties (W: water content, BD: Bulk Density, pH, N: total N content, SOC: organic C content, C/N) and elemental content (P, S, K). Significant P-values are marked in bold.

Soil properties	One-way ANOVA				Tukey's HSD post-hoc					
	DF	SS	F	P	G vs E	G vs I	G vs L	E vs I	E vs L	I vs L
W	3	9.868	6.666	**<0.001**	0.937	0.462	**<0.001**	0.184	**<0.001**	0.195
BD	3	1033	3.946	**0.01**	0.189	0.999	0.533	0.159	**<0.01**	0.589
pH	3	0.294	1.848	0.152	0.999	0.527	0.244	0.538	0.251	0.950
N	3	1.677	15.06	**<0.001**	0.988	0.481	**<0.001**	0.677	**<0.001**	**<0.001**
SOC	3	529.8	10.61	**<0.001**	0.992	0.406	**<0.001**	0.576	**<0.001**	**<0.001**
C/N	3	148.3	7.161	**<0.001**	0.988	0.160	**<0.01**	0.285	**<0.001**	0.211
P	3	5.015	2.686	**0.05**	0.793	0.04	0.372	0.275	0.892	0.682
S	3	1.192	2.193	0.102	0.958	0.746	0.491	0.956	0.233	0.085
K	3	63.69	8.508	**<0.001**	**<0.001**	**<0.001**	0.341	1.000	0.070	0.070

Supplementary Table S2. Pairwise comparisons among microbial community compositions from different afforestation stages. Data refer to P-values of pairwise comparisons (bootstrap tests with 999 permutations) after PERMANOVA (see Table 2 in main text).

	**Bacteria**		**Fungi**	
**Factor**	F	P	F	P
Stage	5.877	**0.001**	6.918	**0.001**
G vs E		0.290		0.947
I vs E		0.436		0.963
L vs E		0.824		0.061
I vs E		0.992		0.735
L vs G		**0.049**		**0.015**
L vs I		0.096		0.167

Supplementary Table S3. **Counts of ASVs and sequences in bacteria and fungi datasets.** Data refer to counts for each sample (identified by chronosequence, CHR, afforestation stage, ST, and replicate, Rep) in bacteria and fungi datasets before (Pre) ad after (Post) the filtering procedure (i.e. elimination of ASVs with abundance lower than 0.1% in all samples). The percentage of retained abundance (i.e. retained sequences) after filtering is also reported.

CHR	ST	Rep	Bacteria (16S)					Fungi (ITS)				
			ASVs		Sequences			ASVs		Sequences		
			Pre	Post	Pre	Post	% retained abundance	Pre	Post	Pre	Post	% retained abundance
1	G	a	17541	544	257079	69911	27.2	7066	613	268335	187964	70.0
1	G	b	15512	567	176897	47857	27.1	7380	626	300684	212250	70.6
1	G	c	19765	617	311858	82598	26.5	8458	692	345757	230331	66.6
1	E	a	25252	879	409314	102853	25.1	8789	622	187047	99995	53.5
1	E	b	19325	816	245578	66930	27.3	8663	582	181500	91728	50.5
1	E	c	16112	545	171538	42324	24.7	9660	602	192827	94877	49.2
1	I	a	20478	725	240868	71766	29.8	10341	582	280401	168387	60.1
1	I	b	21090	733	290093	93973	32.4	9114	599	212570	121250	57.0
1	I	c	22814	733	346323	100392	29.0	8037	533	160332	98962	61.7
1	L	a	12172	734	292923	155494	53.1	4936	505	215152	179695	83.5
1	L	b	15574	766	625491	328010	52.4	5039	481	240614	201175	83.6
1	L	c	9937	715	183283	98573	53.8	4734	467	201959	165266	81.8
2	G	a	19602	668	261323	65167	24.9	6217	566	184272	123404	67.0
2	G	b	21956	674	365601	94230	25.8	5278	565	181393	138361	76.3
2	G	c	18433	686	243655	66708	27.4	7346	568	280333	198801	70.9
2	E	a	17667	697	295314	87541	29.6	8467	575	216513	112807	52.1
2	E	b	15976	664	211913	61152	28.9	6446	587	127938	64878	50.7
2	E	c	15026	647	190652	51858	27.2	5182	495	89334	44304	49.6
2	I	a	16201	794	166648	63499	38.1	5827	586	222178	167425	75.4
2	I	b	16146	742	176612	63331	35.9	5796	584	242931	185996	76.6
2	I	c	19072	793	262043	102756	39.2	5574	582	239236	184580	77.2
2	L	a	13504	667	129900	59694	46.0	3861	477	188670	161713	85.7
2	L	b	26151	762	634513	292115	46.0	5533	618	356805	307580	86.2
2	L	c	16613	710	198207	90706	45.8	5346	558	260769	211066	80.9
3	G	a	19935	668	209181	55405	26.5	6401	598	136962	85987	62.8
3	G	b	24897	687	390985	98054	25.1	6768	578	141220	84493	59.8
3	G	c	20661	680	226815	57602	25.4	7550	573	162767	89492	55.0
3	E	a	17082	634	197879	56971	28.8	10215	686	212785	108371	50.9
3	E	b	19412	609	311707	94358	30.3	7406	575	139203	75745	54.4
3	E	c	20840	619	342705	93057	27.2	8141	615	173340	90106	52.0
3	I	a	16530	709	208102	78054	37.5	6135	553	310690	256430	82.5
3	I	b	17342	750	194142	65682	33.8	5765	476	196757	149203	75.8
3	I	c	17514	697	213357	74530	34.9	6701	530	268453	205189	76.4
3	L	a	14802	528	172572	47009	27.2	5972	472	219447	154617	70.5
3	L	b	17812	582	245054	62799	25.6	6656	542	205467	136080	66.2
3	L	c	16710	562	222359	63477	28.5	6379	529	222571	155657	69.9
4	G	a	19255	683	251732	69321	27.5	7403	627	202475	128677	63.6
4	G	b	21989	646	385433	104679	27.2	5774	581	122920	79058	64.3
4	G	c	18900	616	225731	57191	25.3	5806	566	137158	91346	66.6
4	E	a	25102	820	428707	149942	35.0	6570	625	151233	106989	70.7
4	E	b	19007	772	216334	76400	35.3	5635	558	141766	103446	73.0
4	E	c	19939	780	271775	99330	36.5	7240	637	242029	171971	71.1
4	I	a	18309	791	234181	85440	36.5	4657	522	183913	146962	79.9
4	I	b	19612	760	235170	78773	33.5	4255	538	194123	164258	84.6
4	I	c	24595	795	420843	136364	32.4	3931	571	204279	176777	86.5
4	L	a	19793	674	258111	80028	31.0	3255	461	316478	288849	91.3
4	L	b	17012	703	161151	52205	32.4	4411	597	305510	272101	89.1
4	L	c	18793	649	289608	114766	39.6	3026	481	218514	195631	89.5
**All**	**All**	**All**	**148731**	**1101**	**13001260**	**4310875**	**33.2**	**103169**	**1638**	**10187610**	**7270230**	**71.4**
